# Supplementary figures and images for: Colibactin possessing E. coli isolates in association with colorectal cancer and their genetic diversity among Pakistani population
Source: PLoS One. 2022 Nov 11;17(11):e0262662. doi: 10.1371/journal.pone.0262662 (PMC9651576; doi:10.1371/journal.pone.0262662)

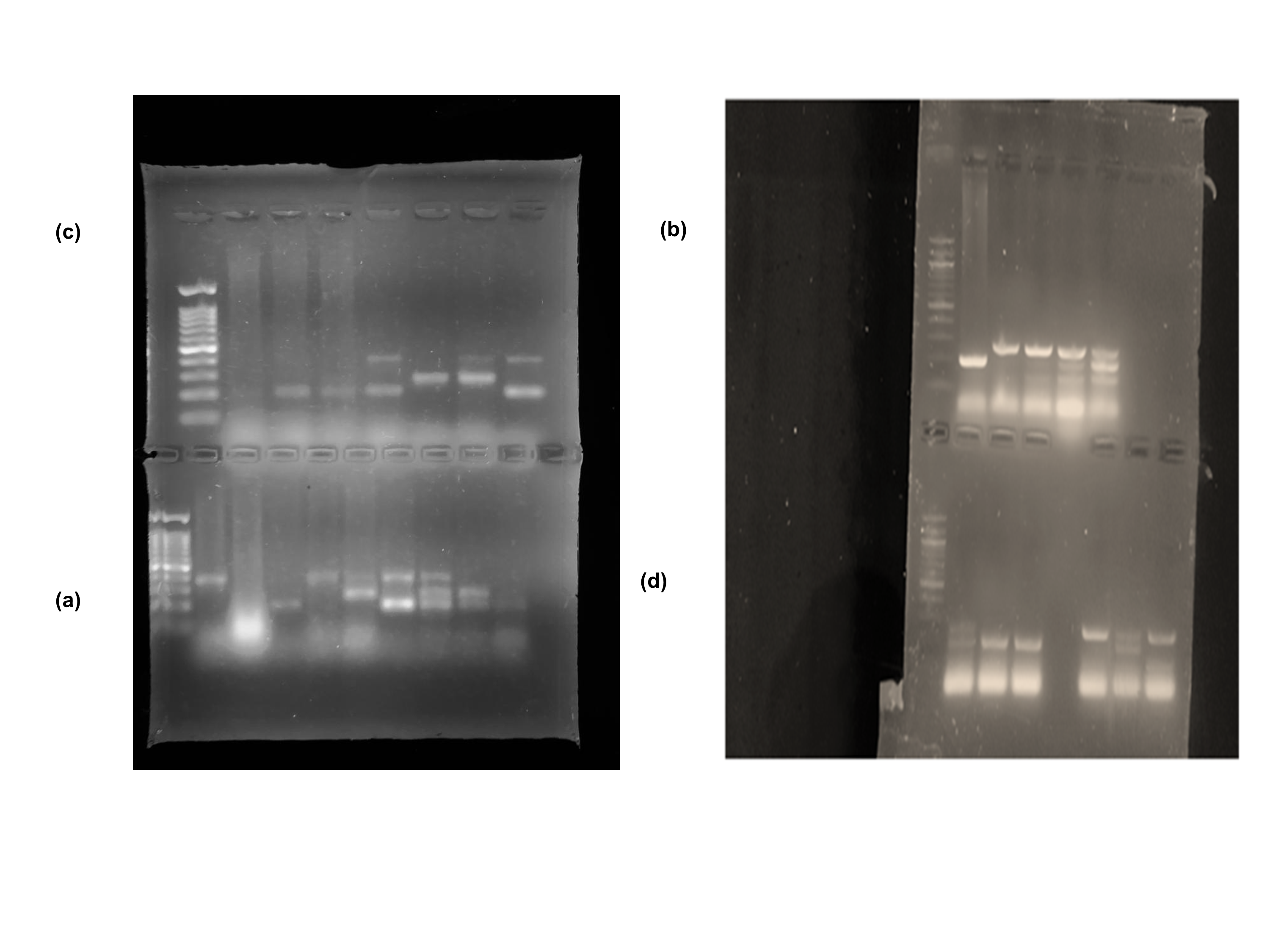

Supplement: S1 Raw images — (TIF) [file pone.0262662.s002.tif]
