# Supplementary material for: Colibactin possessing E. coli isolates in association with colorectal cancer and their genetic diversity among Pakistani population
Source: PLoS One. 2022 Nov 11;17(11):e0262662. doi: 10.1371/journal.pone.0262662 (PMC9651576; doi:10.1371/journal.pone.0262662)
Supplement: S1 Table — (DOCX) [file pone.0262662.s003.docx]

| **Target** | **Primer** | **Primer sequence** | **Product Size (bps)** | **Reference** | **Annealing** |
| --- | --- | --- | --- | --- | --- |
| *Chu*A | *chu*A. 1b | 5’- GAC GAA CCA ACG GTC AGG AT-3’ | 288 | [[23](#_ENREF_23)] | 59 °C |
|  | *chu*A.2 | 5’- TGC CGC CAG TAC CAA AGA CA-3’ |  |  |  |
| *yja*A | yjaA. 1b | 5’-TGA AGT GTC AGG AGA CGC TG-3’ | 211 |  |  |
|  | YjaA.2b | 5’-ATG GAG AAT GCG TTC CTC AAC-3’ |  |  |  |
| *TspE*4.C2 | TspE4C2. 1b | 5’-GAG TAA TGT CGG GGC ATT CA-3’ | 152 |  |  |
|  | TspE4C2. 2b | 5’-CGC GCC AAC AAA GTA TTA CG-3’ |  |  |  |
| *arp*A | Acek.f | 5’-AAC GCT ATT CGC CAG GTT GC-3’ | 400 |  |  |
|  | ArpA1.r | 5’-TCT CCC CAT CCG TAC CGT ACG CGC TA-3’ |  |  |  |
| *ClbA* | ClbA F | 5’ AAGCCGTATCCTGCTCAAAA ‘3 | 342 | [[24](#_ENREF_24)] | 60°C |
|  | ClbA R | 5’ GCTTCTTTGAGCGTCCACAT ‘3 |  |  |  |
| *ClbQ* | ClbQ F | 5’ GCACGATCGGACAGGTTAAT ‘3 | 308 |  |  |
|  | ClbQ R | 5’ TAGTCTCGGAGGGATCATGG ‘3 |  |  |  |
| *ClbB* | ClbB F | 5’ GATTTGGATACTGGCGATAACCG ‘3 | 579 | [[25](#_ENREF_25)] | 60°C |
|  | ClbB R | 5’ CCATTTCCCGTTTGAGCACAC ‘3 |  |  |  |

**Table S1:** **Detailed list of primers used in the study**
