# Supplementary material for: Colibactin possessing E. coli isolates in association with colorectal cancer and their genetic diversity among Pakistani population
Source: PLoS One. 2022 Nov 11;17(11):e0262662. doi: 10.1371/journal.pone.0262662 (PMC9651576; doi:10.1371/journal.pone.0262662)
Supplement: S2 Table — (DOCX) [file pone.0262662.s004.docx]

**Table S2. socio-demographic data of patients was collected using questionnaires.**

| **Questionnaires** | | | | | | | |
| --- | --- | --- | --- | --- | --- | --- | --- |
| **Sr #** | **Parameters** | **Response** | | | | | |
| 1 | Personal Information | Age | Gender | Marital Status | Occupation | Income | Family |
| 2 | Tooth Brushing | Always | Often | Seldom | Never |  |  |
| 3 | Miswak Usage | Always | Often | Seldom | Never |  |  |
| 4 | Salt Intake | No | Low | Normal | High |  |  |
| 5 | Chili Consumption | No | Rarely | Frequently | Moderate |  |  |
| 6 | Dairy Product Use | No | Rarely | Frequently | Moderate |  |  |
| 7 | Black Tea | No | Rarely | Frequently | Moderate |  |  |
| 8 | Green Tea | No | Rarely | Frequently | Moderate |  |  |
| 9 | Eating Fresh Fruits And Vegetables | No | Rarely | Frequently | Moderate |  |  |
| 10 | Eating Rice | No | Rarely | Frequently | Moderate |  |  |
| 11 | Eating Red Meat | No | Rarely | Frequently | Moderate |  |  |
| 12 | Eating Processed Meat Or Food | No | Rarely | Frequently | Moderate |  |  |
| 13 | Eating Sweets | No | Rarely | Frequently | Moderate |  |  |
| 14 | Junk Food Intake | No | Rarely | Frequently | Moderate |  |  |
| 15 | Physical Activity | No | Rarely | Frequently | Moderate |  |  |
| 16 | Washing Hand Before Meal | Always | Often | Seldom | Never |  |  |
| 17 | Hand Washing After Using Toilet | Always | Often | Seldom | Never |  |  |
| 18 | Hand Washing With Soap | Always | Often | Seldom | Never |  |  |
| 19 | Drinking Water Source | City | Well | Boring | Bottled | Filtered |  |
| 20 | Sewage System | Proper | Damaged | Nil |  |  |  |
| 21 | Household Animals | Poultry | Cat | Dogs | Cow | Goat | Parrot |
| 22 | Cockroaches In House | Yes | No |  |  |  |  |
| 23 | House Flies | Yes | No |  |  |  |  |
| 24 | Addiction | Cigarette | Alcohol | Pan/Supari | Niswar | Never | Past |
| 25 | Living With Someone Who Smoke Regularly | Yes | No |  |  |  |  |
| 26 | Medicine Use In Last 15 Days | Antibiotics | PPI | NSAID | Others | Nil |  |
| 27 | Family History Of Cancer | Yes | No |  |  |  |  |
| 28 | Ethinic Background |  |  |  |  |  |  |
| 29 | Type 2 Diabetes | Yes | No |  |  |  |  |
| 30 | Known Allergy To Any Medicine | Yes | No |  |  |  |  |
| 31 | Symptoms | Mild | Moderate | Sever | other | Nil |  |
| 32 | Endoscopy Findings |  |  |  |  |  |  |
| 33 | Colonoscopy Findings |  |  |  |  |  |  |
| 34 | Tumor | Location | Type | Size | Stage | other |  |
